# Specific Absorption Rate Optimization in Microwave Cancer Hyperthermia via Local Power Synthesis Algorithm

**DOI:** 10.3390/cancers17172813

**Published:** 2025-08-28

**Authors:** Maryam Firuzalizadeh, Rossella Gaffoglio, Giorgio Giordanengo, Marco Righero, Giuseppe Vecchi

**Affiliations:** 1 Department of Electronics and Telecommunications, Politecnico di Torino, 10129 Turin, Italy; giuseppe.vecchi@polito.it; 2 Advanced Computing, Photonics & Electromagnetics (CPE) Area, Fondazione LINKS, 10138 Turin, Italy; rossella.gaffoglio@linksfoundation.com (R.G.); giorgio.giordanengo@linksfoundation.com (G.G.); marco.righero@linksfoundation.com (M.R.)

**Keywords:** alternating projections algorithm (APA), microwave hyperthermia (HT), particle swarm optimization (PSO), specific absorption rate (SAR)

## Abstract

Objective: Microwave hyperthermia is a clinically validated adjunctive therapy in oncology, employing antenna applicators to selectively raise tumor tissue temperature to 40–44 °C. For deep-seated tumors, especially those in anatomically complex areas like the head and neck (H&N) region, phased array antennas are typically employed. Determining optimal antenna feeding coefficients is crucial to maximize the specific absorption rate (SAR) within the tumor and minimize hotspots in healthy tissues. Conventionally, this optimization relies on meta-heuristic global algorithms such as particle swarm optimization (PSO). Methods: In this study, we consider a deterministic alternative to PSO in microwave hyperthermia SAR-based optimization, which is based on the Alternating Projections Algorithm (APA). This method iteratively projects the electric field distribution onto a set of constraints to shape the power deposition within a predefined mask, enforcing SAR focusing within the tumor while actively suppressing deposition in healthy tissues. To address the challenge of selecting appropriate power levels, we introduce an adaptive power threshold search mechanism using a properly defined quality parameter, which quantifies the excess of deposited power in healthy tissues. Results: The proposed method is validated on both a simplified numerical testbed and a realistic anatomical phantom. Results demonstrate that the proposed method achieves heating quality comparable to PSO in terms of tumor targeting, while significantly improving hotspot suppression. Conclusions: The proposed APA framework offers a fast and effective deterministic alternative to meta-heuristic methods, enabling SAR-based optimization in microwave hyperthermia with improved tumor targeting and enhanced suppression of hotspots in healthy tissue.

## Simple Summary

This study investigates the application of the Alternating Projections Algorithm (APA) for specific absorption rate (SAR)-based optimization in microwave hyperthermia treatment planning. This method leverages a two-level power mask to iteratively shape the electric field distribution, focusing energy deposition in the tumor region while limiting exposure in healthy tissues. To enhance the effectiveness of this approach, an adaptive mask threshold selection strategy is introduced, based on quantitative metrics that evaluate energy spillover. The performance of APA is assessed using two numerical models of the head and neck (H&N) region: a simplified cylindrical model and a realistic anatomical phantom. Comparative analysis are conducted against the widely used particle swarm optimization (PSO) technique. Results demonstrate that APA achieves comparable tumor coverage while offering superior hotspot suppression, positioning it as a promising deterministic alternative to meta-heuristic SAR optimization strategies.

## 1. Introduction

Microwave hyperthermia (HT) has emerged as a complementary thermal treatment in cancer therapy, demonstrating significant radio- and chemo-sensitizing effects [1]; it consists of increasing the temperature of cancerous tissues to the range of 40–44 °C while minimizing the risk of hotspots in the surrounding healthy tissues [2]. Numerous clinical trials have shown that combining HT with chemotherapy and/or radiotherapy enhances treatment efficacy without exacerbating long-term side effects [1,3,4,5,6,7], with particular effectiveness reported for recurrent cancers [8,9,10].

For sub-superficial and deep-seated tumors, microwave hyperthermia is typically delivered using phased array antenna systems, optimized to provide focused energy on the tumor target through appropriate amplitude and phase settings for each antenna of the applicator [11,12]. Although clinical results show promise, achieving the desired optimal thermal dose for an almost hour-long HT treatment is often hindered by hotspots in normal tissues, which prevent the further increase in total power, impacting the ability to reach the targeted thermal dose [13,14,15].

To address these limitations, hyperthermia treatment planning (HTP) has become an essential step in HT preparation [16]. HTP uses patient-specific anatomical models—derived from CT or MRI scans—and detailed EM simulations to optimize the feeding amplitude and phase of each antenna in the array applicator [17], with the aim of selectively focusing energy within the tumor while limiting exposure in healthy tissues.

Various optimization techniques have been developed for HTP [18,19,20,21,22,23]; among them, specific absorption rate (SAR)-based methods [23,24,25,26,27] remain the most practical and widely adopted technique in clinical settings, typically implemented using global meta-heuristic algorithms such as the particle swarm optimization (PSO) algorithm [28]. While global meta-heuristic algorithms provide robust solutions, they are computationally demanding and may require many iterations to converge. This high computational cost can hinder real-time adjustments or re-optimizations, even though the initial optimization is usually performed during the pre-treatment planning phase.

In recent years, an alternative class of algorithms—local power synthesis techniques—has been explored for SAR-based optimization [29,30]. Inspired by far-field antenna array synthesis [31,32], these methods attempt to directly shape the SAR distribution by constraining the electric field within a spatially defined “power mask”.

In this study, we propose a deterministic SAR-based optimization method based on the Alternating Projections Algorithm (APA), which iteratively enforces convex constraints on the electromagnetic field to confine power deposition within a multi-level spatial mask. This approach enables precise shaping of the SAR pattern, facilitating tumor targeting while actively suppressing energy in healthy tissues.

To overcome the practical challenge of selecting appropriate power thresholds, we introduce an adaptive threshold selection mechanism guided by a SAR-based hotspot suppression indicator, denoted as $V^{H}$, which quantifies the percentage of healthy tissue volume exceeding a relative SAR level.

The proposed APA-based method is validated through simulations in both a simplified numerical testbed and a realistic anatomical model of the H&N region. Its performance is compared to that of the PSO-based SAR optimization in terms of tumor coverage, hotspot suppression, and computational efficiency.

## 2. Materials and Methods

### 2.1. Heating Mechanism in Hyperthermia

In order to clarify the physical principles underlying microwave hyperthermia, we briefly recall the standard mechanisms of electromagnetic (EM) energy deposition in biological tissues [33,34].

The interaction of microwave (MW) fields with biological tissue is typically investigated using a macroscopic model including two key parameters: the relative permittivity $\varepsilon_{r}$ (unitless) and the effective electrical conductivity σ (units of S/m) [35]. The relative permittivity describes the ability to polarize a material subjected to an applied EM field [11]. The effective electrical conductivity embodies all electrical losses in the material due to the currents driven by the EM field, as formalized in well-known macroscopic tissue models (e.g., Cole–Cole, Gabriel–Gabriel models) [36,37,38,39]. Both $\varepsilon_{r}$ and σ are strongly frequency- and tissue-dependent, with comprehensive datasets available in the IT’IS database [40]. The dielectric properties of tumor tissue differ from those of normal tissue, reflecting changes in tissue composition and structure [41,42,43].

In lossy media, power dissipation per unit volume due to time-harmonic EM fields is represented by [44]:

(1) $$\frac{dp_{diss}}{dV}=E\left(r,t\right)\;\cdot \;J\left(r,t\right),$$

where $E=ℜ\{Ee^{j\omega t}\}$ is the electric field (V/m) and $J=ℜ\{Je^{j\omega t}\}$ is the induced current density (A/m^2^). Taking the time average over one period *T*, Equation (1) becomes

(2) $${\langle \frac{dp_{diss}}{dV}\rangle }_{T}=\frac{1}{2}\sigma \left(r\right){\left|E\left(r\right)\right|}^{2},$$

using the macroscopic constitutive relation J=σE (with effective conductivity σ) [35].

The quantity in (2) is the volumetric heat source (W/m^3^) generated by EM absorption and we denote it with $Q_{EM}$:

(3) $$Q_{EM}\left(r\right)=\frac{1}{2}\sigma \left(r\right){\left|E\left(r\right)\right|}^{2}.$$

The convention adopted in Equation (3) assumes complex peak phasors; when RMS fields are used, the prefactor 1/2 is omitted.

A metric widely adopted in MW hyperthermia [35] is the specific absorption rate (SAR) (units of W/kg) which quantifies the rate at which the EM energy is absorbed per unit mass of tissue [45]), defined as

(4) $$\mathrm{SAR}\left(r\right)=\frac{Q_{EM}\left(r\right)}{\rho (r)},$$

so that

(5) $$\mathrm{SAR}\left(r\right)=\frac{1}{2}\frac{\sigma (r)}{\rho (r)}{\left|E\left(r\right)\right|}^{2},$$

where ρ (kg/$m^{3}$) is the tissue mass density, at position vector r.

As common in hyperthermia literature, the rise in tissue temperature caused by the absorption of EM energy can be described using Pennes’ bioheat equation (PBHE) [46]:

(6) $$\rho C_{p}\frac{\partial T}{\partial t}=\nabla \;\cdot \;\left(k\nabla T\right)-\rho_{b}C_{p,b}\omega \rho \left(T-T_{a}\right)+Q_{met}+Q_{EM},$$

where *k* (W/m/°C) is the tissue thermal conductivity, $\rho_{b}=1060$$\mathrm{kg}/m^{3}$ and $C_{p,b}=3890$ J/kg/°C are the volume density and specific heat capacity of blood, $T_{a}=37$ °C is the arterial blood temperature, and ω (mL/min/kg) is the tissue volumetric blood perfusion rate. Moreover, $Q_{EM}$ is the EM heat source related to SAR (Equation (4)) and $Q_{met}$ is the metabolic heat generation term (often negligible w.r.t. external heat sources).

While PBHE is widely adopted for treatment planning due to its simplicity, more detailed models (e.g., discrete vasculature models [47]) provide improved accuracy but remain computationally demanding.

In summary, tissue heating under MW exposure arises from fundamental electrodynamic principles: effective conductivity captures both conduction and dielectric losses in a macroscopic sense. The SAR metric provides the standard bridge between EM fields and thermal response, and—combined with bioheat modeling—constitutes the accepted theoretical foundation for clinical hyperthermia.

### 2.2. Optimization Approach

The primary objective of SAR-based optimization is to maximize power deposition within the tumor region while minimizing the risk of overheating in the surrounding healthy tissues. In deep hyperthermia, the electromagnetic field must inevitably propagate through layers of healthy tissue before reaching the tumor—this is precisely what makes selective heating challenging. The optimization procedure adjusts the feeding coefficients of the antenna array to exploit constructive interference in the tumor and destructive interference elsewhere, thereby focusing the absorbed power where it is needed.

The total electric field can be written as the superposition of the individual electric fields generated by each antenna element when excited individually, weighted by their corresponding complex excitation coefficients. Accordingly,

(7) $$E\left(r\right)=\sum_{n=1}^{N}b_{n}\;e_{n}\left(r\right),$$

where $e_{n}\left(r\right)$ denotes the electric field at point r generated by the *n*th antenna with unit excitation (all others turned off), and $b_{n}$ is the complex excitation coefficient (amplitude and phase) of the *n*th antenna in an *N*-element array. In the present work, we considered an array of patch antennas [48], linearly polarized along the vertical (*z*) axis, as common in deep hyperthermia systems [49].

To optimize the excitation coefficients [b], we apply the Alternating Projections Algorithm (APA). We discretize the electric fields defined in Equation (7) over a set of *M* sampling points $r_{m}$, corresponding to the mesh points in the simulated volume of interest. The matrix,

(8) $$\left[E_{N}\right]=\left[\left[e_{1}\right],\left[e_{2}\right],\ldots ,\left[e_{N}\right]\right],$$

collects the simulated electric fields generated by each unit-excited antenna. The field distributions are calculated only once at the beginning of the optimization. These fields are obtained from full-wave simulations in a heterogeneous anatomical model, where each tissue is assigned its own dielectric properties. Each column, $\left[e_{n}\right]$, n=1,…,N, contains the values of the electric field vector $e_{n}\left(r\right)$ estimated in the set of spatial points $r_{m}$, m=1,…,M, arranged by stacking the three Cartesian components as follows:

(9) $$\left[e_{n}\right]=\left[\begin{array}{c} \left[e_{n,x}\right]\\ \left[e_{n,y}\right]\\ \left[e_{n,z}\right] \end{array}\right],$$

with

(10) $$\left[e_{n,k}\right]=\left[\begin{array}{c} e_{n,k}\left(r_{1}\right)\\ \vdots \\ e_{n,k}\left(r_{M}\right) \end{array}\right],\;k=x,y,z.$$

To reproduce a power pattern focused on the tumor mass, a starting target field $\left[E_{t}\right]$ (e.g., a Gaussian field centered on the tumor region) is considered and projected onto the column space of the matrix $\left[E_{N}\right]$ to find a reconstructed field $\left[E_{t}^{rec}\right]$. Before generating the initial target field $\left[E_{t}\right]$, we assess the polarization of the unit-excited fields $e_{n}$ by examining the dominant component in the matrix $\left[E_{N}\right]$. In our setup, the *z*-component entries are about one order of magnitude larger than the *x*- and *y*-components, reflecting the intrinsic polarization of the chosen antenna type. The Gaussian target $\left[E_{t}\right]$ is then generated using this dominant component, ensuring that its polarization matches that of $e_{n}$. The amplitude and width of this starting Gaussian target field were properly fixed to fit an optimized power distribution in the region of interest. Since the first reconstructed field was considerably different from the target field, an iterative procedure was implemented to adapt its profile to the desired focused pattern.

**Figure 1:**
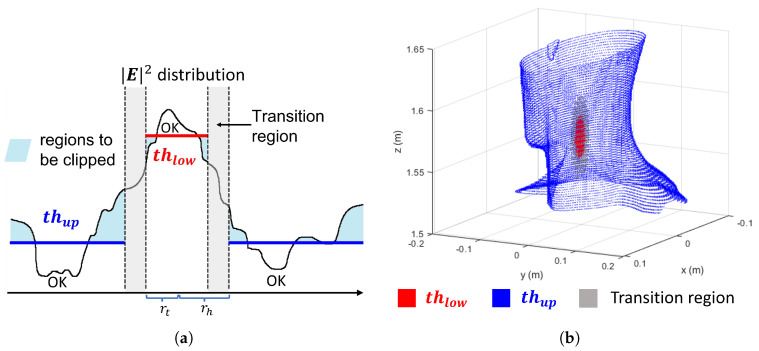
(**a**) Clipping of a generic 1D function using a two-level mask, where values are confined between two thresholds $th_{low}$ and $th_{up}$ through smooth transitions. The distances $r_{t}$ and $r_{h}$ are the maximum radial distances from the tumor center that define, respectively, the boundary of the tumor region and the outer edge of the transition (gray) region. (**b**) APA mask defined on a 3D phantom: red points correspond to the tumor region where the $th_{low}$ is applied; gray points denote the transition region where no constraints are imposed; blue points indicate the healthy region (outside the transition zone) where the $th_{up}$ is enforced. For visualization clarity, only the boundary of the healthy region is shown in blue.

First, following common HTP procedures, the tumor (T) and healthy (H) tissue regions are identified; this step is performed in clinical practice using patient-specific anatomical segmentations obtained from CT or MRI scans. Then, at each step of this procedure, the squared norm of the reconstructed field is compared to a power mask with two threshold levels (see for reference Figure 1 and Section 3.2 for details). To ensure a sufficient field focusing on the target, the first level $th_{low}$ is enforced within the tumor tissue T, defined as a region with maximum radial extent $r_{t}$. To avoid hotspots, the second level $th_{up}$ is applied in the healthy tissue H located outside $r_{h}$, where $r_{h}$ denotes the outer edge of the transition region measured radially from the tumor centroid. The transition region is thus a shell region defined between $r_{t}$ and $r_{h}$ where no constraints are imposed, allowing for a more gradual tapering of the field (see Figure 1b).

When the squared modulus of the reconstructed field is below $th_{low}$ for $r_{m}\in T$, or exceeds $th_{up}$ for $r_{m}\in H$, its value is adjusted (clipped) to the corresponding mask threshold, and the new field is projected again onto the columns of $\left[E_{N}\right]$, providing a vector of complex antenna feedings $b_{n}$, n=1,…,N, which is normalized at each step to fix the total input power to 1 W. This normalization includes the parameters $P_{0}$, the total power fed to the array, $R_{0}$, the reference input resistance, and $∥\left[b\right]{∥}^{2}$, the squared norm of the complex excitation vector.

The detailed steps of the APA optimization procedure are outlined in Algorithm 1.
**Algorithm 1** APA Optimization Algorithm  1:**procedure** APA Optimization  2:    **Inputs:**    Electric field matrix $\left[E_{N}\right]$, Gaussian initial guess $\left[E_{t}\right]$, tumor region T, healthy region H, $th_{low}$, $th_{up}$, input power $P_{0}$, reference input resistance $R_{0}$  3:    **Output:**    Optimized antenna excitation vector $\left[\tilde{b}\right]$  4:    **Initialization:**  5:    Compute pseudo-inverse: ${\left[E_{N}\right]}^{†}=\mathrm{pinv}\left(\left[E_{N}\right]\right)$  6:    $\left[b\right]={\left[E_{N}\right]}^{†}\;\cdot \;\left[E_{t}\right]$  7:    $C=P_{0}/{(∥\left[b\right]∥}^{2}/\left(2R_{0}\right))$  8:    $\left[\tilde{b}\right]=\sqrt{C}\;\left[b\right]$  9:    $\left[E_{t}^{rec}\right]=\left[E_{N}\right]\;\cdot \;\left[\tilde{b}\right]$10:    **while** stopping criterion not met **do**11:        **for** each voxel $r_{m}\in T$ **do**12:           **if** $|{\left[E_{t}^{rec}\right]}_{m}{|}^{2}<th_{low}$ **then**13:               ${\left[E_{t}^{rec}\right]}_{m}=\sqrt{th_{low}}\;\cdot \;\frac{{\left[E_{t}^{rec}\right]}_{m}}{|{\left[E_{t}^{rec}\right]}_{m}|}$14:           **end if**15:        **end for**16:        **for** each voxel $r_{m}\in H$ **do**17:           **if** $|{\left[E_{t}^{rec}\right]}_{m}{|}^{2}>th_{up}$ **then**18:               ${\left[E_{t}^{rec}\right]}_{m}=\sqrt{th_{up}}\;\cdot \;\frac{{\left[E_{t}^{rec}\right]}_{m}}{|{\left[E_{t}^{rec}\right]}_{m}|}$19:           **end if**20:        **end for**21:        $\left[b\right]={\left[E_{N}\right]}^{†}\;\cdot \;\left[E_{t}^{rec}\right]$22:        $C=P_{0}/{(∥\left[b\right]∥}^{2}/\left(2R_{0}\right))$23:        $\left[\tilde{b}\right]=\sqrt{C}\;\left[b\right]$24:        $\left[E_{t}^{rec}\right]=\left[E_{N}\right]\;\cdot \;\left[\tilde{b}\right]$25:    **end while**26:**end procedure**

To monitor convergence and determine the stopping point of the iterative process, we adopt a well-established criterion from the literature [23], aimed at minimizing the Hotspot-to-Target SAR Quotient (HTQ), defined as

(11) $$\mathrm{HTQ}=\frac{\langle {\mathrm{SAR}}_{\;V1}\rangle }{\langle {\mathrm{SAR}}_{\;\mathrm{TARGET}}\rangle },$$

where $\langle {\mathrm{SAR}}_{\;V1}\rangle$ is the average SAR in V1, with V1 being the 1% of the healthy volume with the highest SAR [50,51], and $\langle {\mathrm{SAR}}_{\;\mathrm{TARGET}}\rangle$ is the average SAR in the target region. It should be noted that, for the evaluation of the HTQ metric, the healthy tissue region is defined to include both the outer healthy region H and the transition region (see Figure 1), i.e., all tissues outside the tumor, in accordance with the standard definition [50].

The developed APA algorithm includes a dynamic stopping criterion based on the evolution of the HTQ parameter and the number of field points exceeding the power mask. Specifically, the optimization is stopped when the HTQ reaches a plateau, or begins to increase, and when the number of out-of-mask points starts increasing persistently indicating that no further meaningful improvement can be achieved. This safeguards against unnecessary iterations and ensures timely convergence within a maximum of 4000 iterations.

## 3. Results

### 3.1. Reference Testbed

**Figure 2:**
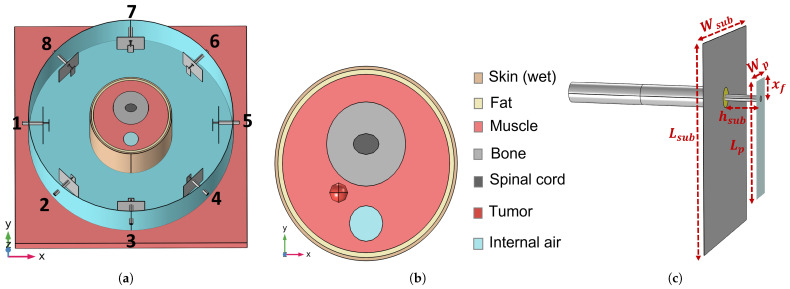
(**a**) Circular array of patch antennas surrounding the simple neck model immersed in the waterbolus. (**b**) Middle-top view (z=0 plane) of the simplified neck model with all the considered tissues. (**c**) Employed patch antenna and optimized dimensions.

To validate the APA-based SAR optimization, a simplified numerical phantom of the human neck and an HT applicator were modeled in COMSOL Multiphysics^®^ (Stockholm, Sweden)  [52]. The setup, illustrated in Figure 2a, consists of a cylindrical phantom mimicking the human neck, surrounded by a circular array of N=8 patch antennas, and immersed in water to emulate the clinically used waterbolus [49]. This device is a plastic bag filled with circulating demineralized water to enhance electromagnetic coupling into tissues, preventing superficial heating, and improve patient comfort [49].

The neck phantom is composed of concentric tissue layers, each assigned electromagnetic properties representative of typical H&N anatomy, following the widely accepted dielectric model for biological tissues [38,39,40]. As illustrated in Figure 2b, the tissues include skin, fat, muscle, bone, spinal cord, and a tumor region. The tumor is modeled as a sphere with radius $r_{t}=6$ mm and is embedded within the phantom at coordinates (−18,−18,−15) mm relative to the center of the cylindrical phantom. For the tumor tissue, dielectric properties have been tailored for realistic H&N HTP according to [41] ($\varepsilon_{r}=59$ and σ=0.89 S/m). Moreover, to mimic the influence of the shoulders and minimize air–water boundary effects, the phantom is positioned on a 4 cm thick muscle layer [48]. For APA optimization, the outer radius of the transition region was assumed to be almost 1 cm greater than the extent $r_{t}$ of the tumor, i.e., $r_{h}=18$ mm [53].

The array of patch antennas was designed and optimized to operate efficiently at the frequency f=434 MHz, as commonly used in clinical hyperthermia systems [48]. With reference to Figure 2c, the optimized antenna geometry includes a ground plane of dimensions $L_{sub}=50$ mm, $W_{sub}=40$ mm. The patch is placed at a distance $h_{sub}=8.8$ mm from the ground and has dimensions $L_{p}=28.8$ mm and $W_{p}=8.4$ mm. The antenna is fed by a coaxial probe located at an offset distance $x_{f}=4.8$ mm from the patch edge.

In the implemented model, the electromagnetic and thermal distributions are computed using the finite element method (FEM) [52]. A tetrahedral mesh is employed, with maximum element sizes of 5 mm in the neck tissues and 1 mm in the antenna applicator, as chosen to find a suitable trade-off between computational cost and solution accuracy [2].

### 3.2. Power Levels Search

The effectiveness of the APA in maximizing power deposition in the tumor region while suppressing unwanted hotspots in healthy tissues depends critically on the proper selection of the power mask thresholds. As illustrated in Section 2.2, the first threshold $th_{low}$ is applied in the tumor region T to ensure sufficient field focusing, while the second threshold $th_{up}$ is defined in the healthy tissues H to suppress the formation of hotspots. A transition region between these levels allows smooth clipping, avoiding abrupt constraint changes in the field pattern (see Figure 1a).

To identify suitable values for these thresholds for the simplified testbed described in Section 3.1, we introduce a metric denoted as $V_{\chi \%}^{H}$, which measures the percentage of healthy tissue volume experiencing SAR values above χ% of the maximum SAR within the tumor. The $V_{\chi \%}^{H}$ metric, computed for different χ values, provides a more detailed understanding of SAR distribution than scalar performance metrics such as the HTQ. Two values of this metric are primarily used: $V_{10\%}^{H}$ to assess overall SAR spillover into healthy tissues, and $V_{50\%}^{H}$ to highlight the presence of high-intensity hotspots.

The adaptive power mask search starts with generating a Gaussian field initial guess as detailed in the Algorithm 2. A fixed peak amplitude $A_{0}=10^{4}\;V^{2}/m^{2}$ is imposed at the tumor center $\left(x_{t},y_{t},z_{t}\right)$, ensuring a predefined maximum intensity at the focal point. An upper bound to this quantity may be obtained by evaluating the values of squared modulus of the electric field that can be reached inside the tumor region by each field $e_{n}$ in (7), combining these values assuming complete constructive interference and taking into account the bound on the total input power (lines 7 and 8, as well as 22 and 23, of Algorithm 1).
**Algorithm 2** Adaptive Threshold Search for APA Optimization  1:**Inputs:**    Electric field matrix $\left[E_{N}\right]$, input power $P_{0}$, reference input resistance $R_{0}$, tumor region T with radius $r_{t}$, healthy region H with radius $r_{h}$  2:Fixed Gaussian peak amplitude $A_{0}$ at tumor center $\left(x_{t},y_{t},z_{t}\right)$ with the maximum achievable intensity  3:Candidate set $\left\{th_{up}^{\left(i\right)}\right\}$ to control hotspot suppression  4:**Outputs:**    Optimal excitation vector ${\left[\tilde{b}\right]}^{★}$ and selected threshold $th_{up}^{★}$  5:Determine the dominant polarization component ν of $\left[E_{N}\right]$  6:**for** each $th_{up}^{\left(i\right)}$ in candidate set **do**  7:    Compute standard deviation $\sigma_{0}=r_{h}{\left(2\log\left(A_{0}/th_{up}^{\left(i\right)}\right)\right)}^{-1/2}$  8:    Compute $th_{low}^{\left(i\right)}=A_{0}\;\cdot \;\exp\left(-r_{t}^{2}/\left(2\sigma_{0}^{2}\right)\right)$  9:    Construct Gaussian field $\left[E_{t}\right]$ with parameters $A_{0}$ and $\sigma_{0}$ and polarization ν10:    Run APA Optimization (Algorithm 1) with $\left(\left[E_{t}\right],th_{low}^{\left(i\right)},th_{up}^{\left(i\right)}\right)$11:    Compute metrics $V_{10\%}^{H}$ and $V_{50\%}^{H}$12:    Store results in $Q(th_{up}^{\left(i\right)},V_{10\%}^{H},V_{50\%}^{H})$13:**end for**14:Identify $th_{up}^{★}$ via Pareto trade-off between $V_{10\%}^{H}$ and $V_{50\%}^{H}$15:Return corresponding excitation vector ${\left[\tilde{b}\right]}^{★}$

For each candidate upper threshold $th_{up}^{\left(i\right)}$, which controls hotspot suppression in the healthy tissues, the standard deviation $\sigma_{0}$ of the Gaussian distribution is computed so that the field intensity at the end of the transition region (distance $r_{h}$ from the tumor center) corresponds to $th_{up}^{\left(i\right)}$, i.e., $\sigma_{0}=r_{h}{\left(2\log\left(A_{0}/th_{up}^{\left(i\right)}\right)\right)}^{-1/2}$. The corresponding threshold $th_{low}^{\left(i\right)}$, applied within the tumor region of radius $r_{t}$, is then derived from the same Gaussian distribution as $th_{low}^{\left(i\right)}=A_{0}\;\cdot \;\exp\left(-r_{t}^{2}/\left(2\sigma_{0}^{2}\right)\right)$.

Once the mask parameters $\left(th_{low}^{\left(i\right)},\;th_{up}^{\left(i\right)}\right)$ are defined, the APA optimization (Algorithm 1) is performed, and the resulting SAR distribution is assessed using the $V_{10\%}^{H}$ and $V_{50\%}^{H}$ metrics. These two metrics capture, respectively, the volume of healthy tissue exposed to moderate and high levels of SAR relative to the tumor maximum.

**Figure 3:**
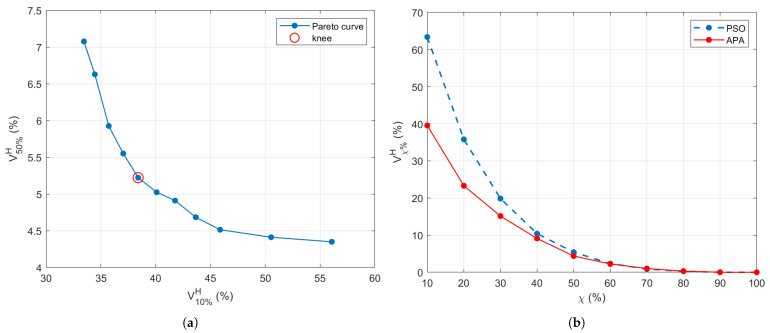
(**a**) Pareto front generated during the APA threshold selection process, showing the trade-off between SAR confinement ($V_{10\%}^{H}$) and hotspot suppression ($V_{50\%}^{H}$). The knee point (highlighted in red) indicates the optimal point, corresponding to $\left(th_{low},\;th_{up}\right)=\left(9.08,\;4.2\right)\times 10^{3}\;V^{2}/m^{2}$. (**b**) Comparison of the $V_{\chi \%}^{H}$ performance across increasing χ% for the APA (using the thresholds corresponding to the knee point) and PSO-based SAR optimization.

A natural trade-off emerges: decreasing $th_{up}$ suppresses hotspots but may also reduce tumor coverage, while increasing it enhances energy delivery to the tumor at the expense of greater off-target exposure. To resolve this, a Pareto front is built by plotting all candidate solutions in the $\left(V_{10\%}^{H},\;V_{50\%}^{H}\right)$ space, and the final configuration is chosen from the knee of the curve—where both objectives are reasonably balanced (see Figure 3a). The knee point (highlighted in red) indicates the optimal point, which corresponds to $\left(th_{low},\;th_{up}\right)=\left(9.08,4.2\right)\times 10^{3}\;V^{2}/m^{2}$. The corresponding APA excitation vector ${\left[\tilde{b}\right]}^{★}$ is retained as the optimal solution. It has been verified that varying the most critical parameters involved in the adaptive threshold search, such as the amplitude $A_{0}$ and the transition region radius $r_{h}$, by 10% of the selected values did not change the achieved results. This indicates a fair stability of the implemented optimization process.

### 3.3. SoA Comparison

To assess the effectiveness of the proposed APA, we compare its performance with that of a conventional PSO-based SAR optimization, a widely used state-of-the-art (SoA) meta-heuristic method. The PSO algorithm is implemented with the same testbed (detailed in Section 3.1) with the goal of focusing the SAR within the tumor while reducing energy deposition in the surrounding healthy tissues by minimizing the cost function HTQ defined in (11).

Figure 3b illustrates the $V_{\chi \%}^{H}$ curves for the PSO benchmark and APA with the selected threshold configuration (corresponding to the knee point of Figure 3a). It is evident that APA shows improved spatial control, with lower SAR leakage into healthy tissues across multiple χ values. This trend confirms that the threshold-guided adaptation in APA yields more consistent control over energy distribution than the global optimization behavior of PSO.

The optimized complex excitation coefficients (amplitudes and phases) for each antenna element resulting from APA and PSO optimization methods are summarized in Table 1. These values represent the final configuration used to drive the antenna array for each method.

From a computational standpoint, the PSO algorithm (with a swarm size of 100) required approximately 140 s to converge on a Intel Core i9 workstation (Intel, Santa Clara, CA, USA) with 128 GB RAM. In contrast, a single APA optimization run was completed in less than 10 s. Including the time required for the adaptive threshold selection procedure (approximately 120 s), the overall APA computational time remains comparable to that of PSO. Notably, the APA achieves superior hotspot suppression while maintaining comparable computational performance. Note that the time required to compute the electromagnetic field matrices—which is common to both methods—is approximately 23 min and not included in the optimization timings. Table 2 summarizes the key properties of the APA and PSO optimizations, including the number of iterations, the total computational time, and the final HTQ values.

**Figure 4:**
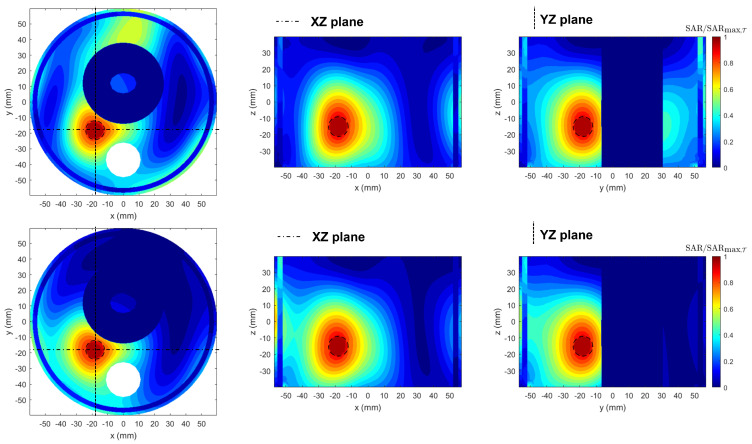
Normalized SAR maps comparison between PSO (**upper row**) and APA (**lower row**), displayed on the three canonical planes cutting the tumor sphere at its centroid. The black dashed circle indicates the tumor sphere region. The APA thresholds considered are $\left(th_{low},\;th_{up}\right)=\left(9.08,4.2\right)\times 10^{3}\;V^{2}/m^{2}$. While both methods achieve good tumor coverage, the APA solution results in more confined SAR patterns, reducing hotspots outside the target.

Qualitative results from the normalized SAR maps—with respect to the maximum SAR within the tumor region (T)—for a given input power are reported in Figure 4. While both PSO and APA provide good target heating, APA offers a tighter confinement of the high-SAR regions, minimizing off-target exposure. This reflects the benefits of using adaptive power thresholding during optimization. Together, these results indicate that APA not only achieves comparable tumor targeting performance to PSO but also improves safety by suppressing undesired SAR levels in the surrounding healthy tissues.

To obtain the temperature distributions, a thermal simulation was performed in COMSOL Multiphysics by solving the heat transfer study based on Pennes’ bioheat equation [46], where the locally computed SAR served as the electromagnetic power deposition source term (see Section 2.1). As common in HTP, we considered the steady-state version of the bioheat equation (Equation (6)), which implies a vanishing time-dependent term (∂T/∂t) [2].

The model incorporated tissue-specific thermal properties and applied convective boundary conditions to mimic heat exchange with the surrounding environment [41,54]. A convective heat flux boundary condition was applied at the interface between the neck and the waterbolus, using a heat transfer coefficient of h=82 W/(m^2^·°C) and an external reference temperature of $T_{\mathrm{ext}}=20$ °C. Additionally, a convective boundary condition was imposed on the internal surface of the trachea, with h=50 W/(m^2^·°C) and $T_{\mathrm{ext}}=30$ °C. The initial tissue temperature was uniformly set to $T_{\mathrm{in}}=37$ °C.

**Figure 5:**
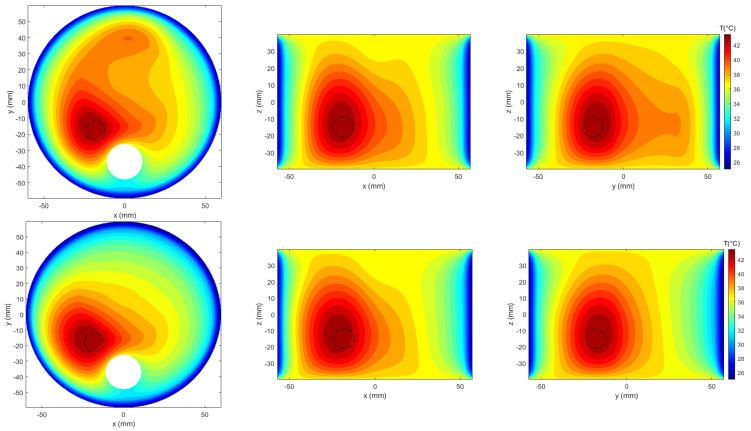
Temperature map comparison between PSO (**upper row**) and APA (**lower row**), visualized on the three canonical planes intersecting the tumor centroid. The maps correspond to the SAR distributions shown in Figure 4, obtained with $P_{0}=26$ W for PSO and $P_{0}=16$ W for APA.

The corresponding temperature maps in Figure 5 further illustrate that APA achieves comparable tumor heating to PSO with a lower total input power ($P_{0}=16$ W vs. $P_{0}=26$ W). These power levels were selected to ensure that the target temperature of 42 °C was reached within the tumor region. The reduced power requirement in the APA case highlights the algorithm’s superior ability to localize energy deposition more effectively within the tumor, thereby improving treatment efficiency while minimizing off-target heating in surrounding healthy tissues.

To demonstrate that the selected power levels ensure adequate thermal coverage, Table 3 reports the standard thermal estimators T90, T50, and T10, defined as the temperature exceeded by 90%, 50%, and 10% of points in the tumor region [2]. The bottom line of Table 3 refers to the case where the feeding coefficients optimized with the APA approach (Table 1) are used in a COMSOL model with the maximum mesh size reduced from 5 mm to 2 mm in the all the neck tissues. A variation of less than 0.2% between values reported in the top and bottom lines of the table demonstrates the robustness of the mesh used to perform the optimization (Section 3.1).

### 3.4. Realistic Testbed

**Figure 6:**
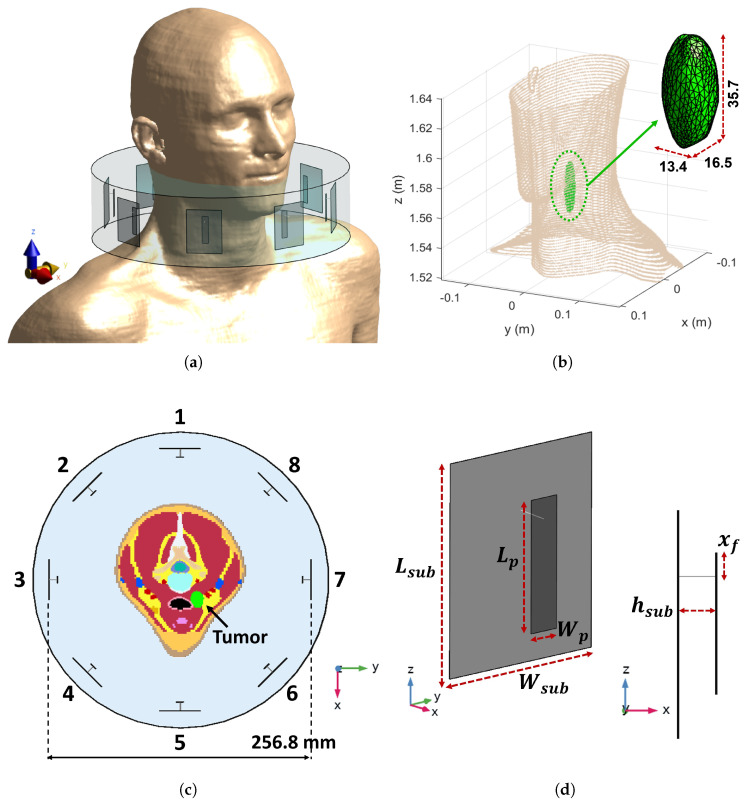
(**a**) Circular array of patch antennas surrounding the neck of the realistic phantom Duke V3.0 [56] and immersed in the waterbolus. (**b**) Tumor target size (expressed in mm) and its position near to the larynx of Duke. (**c**) Top view of the phased array applicator surrounding the segmented phantom visualized on the xy plane; the reported number are used to index the antennas of the array; the tumor target is highlighted in green. (**d**) Employed patch antenna and optimized dimensions.

To evaluate the performance of the APA-based optimization under anatomically realistic conditions, we also considered an HT setup implemented in the Sim4Life simulation platform (Zurich Med Tech AG, Zurich, Switzerland) [55], as illustrated in Figure 6a. The numerical model is based on the phantom Duke V3.0 [56], a member of the Virtual Population (ViP), which was developed from high-resolution magnetic resonance imaging (MRI) scans of healthy volunteers. The phantom includes 305 anatomically segmented tissues, offering a comprehensive and physiologically accurate representation for electromagnetic and thermal simulations.

A tumor region with irregular geometry—approximated as a deformed prolate spheroid—was inserted into the laryngotracheal area (centroid at $x_{t}=18.02$ mm, $y_{t}=16.15$ mm, $z_{t}=1574.9$ mm), as shown in Figure 6b. This placement enables the evaluation of SAR deposition performance in a clinically relevant scenario where anatomical heterogeneity presents significant targeting challenges.

The treatment applicator consists of a uniform circular array of N=8 patch antennas, optimized to operate at f=434 MHz. While based on the same design principles as in the simplified testbed, the antenna dimensions were re-optimized to fit the anatomical features of the realistic phantom. The design parameters of each antenna, shown in Figure 6d, include the following: $L_{sub}=50$ mm, $W_{sub}=40$ mm, $L_{p}=31.0$ mm, $W_{p}=7.2$ mm, $h_{sub}=8.4$ mm, and $x_{f}=4.96$ mm. As in the previous setup, a waterbolus surrounds the applicator (see Figure 6c) [49].

In the Sim4Life model, the electromagnetic and thermal simulations are performed using the finite-difference time-domain (FDTD) method. A rectilinear mesh is employed, with a uniform voxel size of 2 mm in the phantom tissues and 1 mm in the antenna applicator, in compliance with the ESHO guidelines [2].

**Figure 7:**
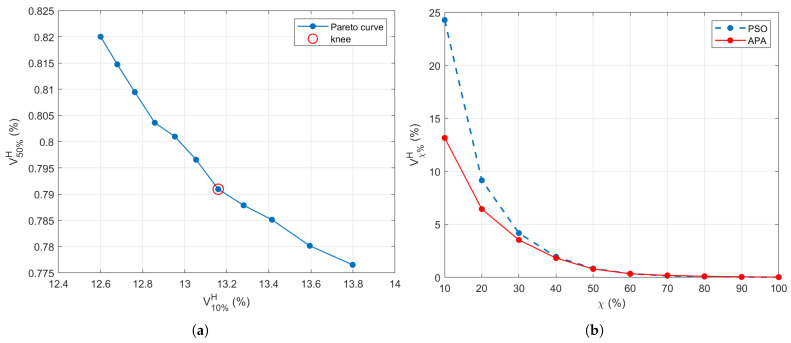
(**a**) Pareto front generated during the APA threshold selection process, showing the trade-off between SAR confinement ($V_{10\%}^{H}$) and hotspot suppression ($V_{50\%}^{H}$). The knee point (highlighted in red) indicates the optimal point, corresponding to $\left(th_{low},\;th_{up}\right)=\left(9.41,\;5.8\right)\times 10^{3}\;V^{2}/m^{2}$. (**b**) Comparison of the $V_{\chi \%}^{H}$ performance across increasing χ% for the APA (using the thresholds corresponding to the knee point) and PSO-based SAR optimization.

To apply the proposed APA, as described in Section 2.2, to this anatomically realistic testbed, the two thresholds must be defined according to the procedure outlined in Section 3.2, which provided the Pareto front displayed in Figure 7a. For this setup, the thresholds were found to be $\left(th_{low},\;th_{up}\right)=\left(9.41,5.8\right)\times 10^{3}\;V^{2}/m^{2}$ for the mask shown in Figure 1b. The $V_{\chi \%}^{H}$ indicator reported in Figure 7b for the APA method, using the selected thresholds, shows an overall better SAR confinement than the PSO. The lower values achieved by the $V_{\chi \%}^{H}$ parameter in Figure 7b with respect to Figure 3b are due to the fact that the defined indicator is a relative parameter expressed over the total number of points in the healthy region, which is significantly greater in the realistic case (1.13 M points) with respect to the simplified model (56 k points). For the realistic model, the computational time required by the APA, including the search algorithm, was less than 80 min as in the case of the PSO algorithm applied here for comparison.

**Figure 8:**
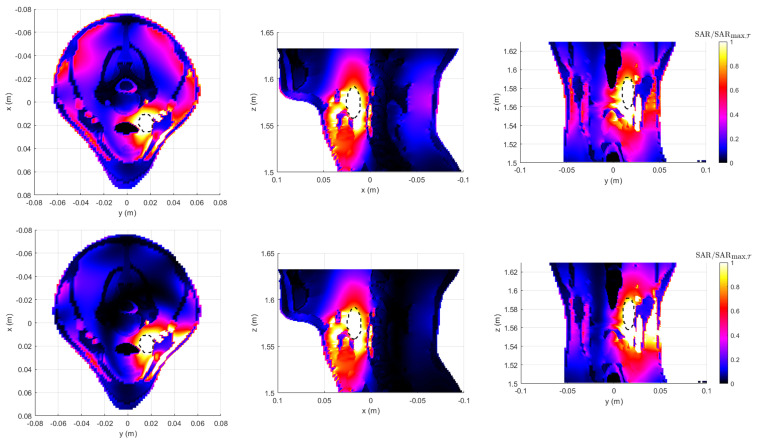
Normalized SAR maps comparison between PSO (**upper row**) and APA (**lower row**), displayed on the three canonical planes cutting the tumor sphere at its centroid. The black dashed line indicates the contour of the target tumor. The APA thresholds considered are $\left(th_{low},\;th_{up}\right)=\left(9.41,5.8\right)\times 10^{3}\;V^{2}/m^{2}$. While both methods achieve good tumor coverage, the APA solution results in more confined SAR patterns, reducing hotspots outside the target.

The resulting normalized SAR distributions—with respect to the maximum SAR within the tumor region (T)—for a given input power are shown in Figure 8. The maps, displayed on the three canonical planes intersecting the tumor centroid (marked by the green dot), compare PSO (upper row) and APA (lower row). While both methods achieve adequate energy focusing on the tumor region, the APA results exhibit a more spatially confined SAR pattern with significantly reduced hotspots in healthy tissues. This improvement arises from APA’s ability to enforce adaptive spatial constraints through the use of a two-level power mask, which simultaneously enhances energy focusing on the tumor and suppresses power deposition in surrounding healthy regions.

Similar to the simplified testbed, the thermal response in the realistic anatomical model was simulated by solving the Pennes’ bioheat equation [46] in Sim4Life, incorporating tissue-specific thermal properties and perfusion rates [40] and completed by setting the boundary conditions as performed in Section 3.3 for the simplified phantom.

**Figure 9:**
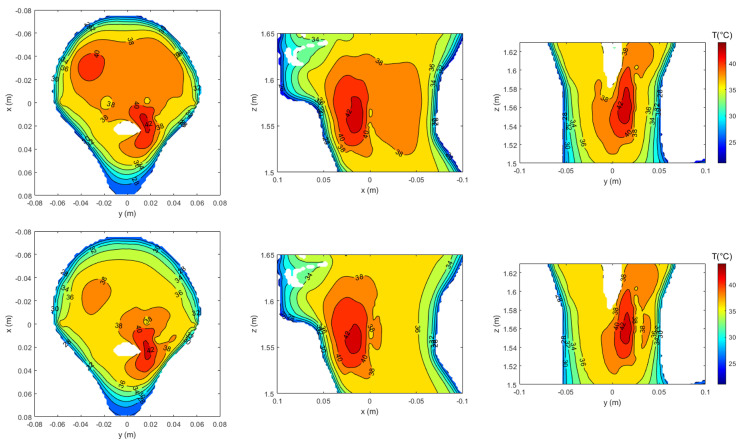
Temperature map comparison between PSO (**upper row**) and APA (**lower row**), visualized on the three canonical planes intersecting the tumor centroid. The maps correspond to the SAR distributions shown in Figure 8, obtained with $P_{0}=60$ W for PSO and $P_{0}=44$ W for APA. Thanks to better energy focusing, APA achieves comparable tumor heating with lower input power, while also reducing the presence of hotspots in healthy tissues.

To further validate these results, the corresponding temperature distributions are illustrated in Figure 9. The PSO algorithm required $P_{0}=60$ W to achieve effective heating (≥42 °C in the tumor region), whereas the APA method reached comparable temperature levels with only $P_{0}=44$ W. This reduction in input power demonstrates the efficiency of APA in shaping the energy deposition more effectively, thus enhancing patient safety and minimizing the risk of overheating in the surrounding healthy tissues. The temperature maps also clearly reflect reduced hotspot formation in the APA case, aligning well with the SAR confinement observed in Figure 8.

To further assess the thermal coverage achieved in the realistic testbed, Table 4 reports the same standard thermal estimators T90, T50, and T10, previously introduced in Section 3.3. These indicators confirm that both the PSO and APA methods deliver effective heating to the tumor region.

## 4. Discussion and Future Works

While the present work focuses on demonstrating the APA optimization approach for deep hyperthermia in the H&N region—a particularly challenging anatomical site due to its complex geometry and heterogeneous tissue composition—the methodology presented is not limited to this application. The same optimization framework can be extended to other anatomical sites commonly treated with deep hyperthermia, such as the pelvic region for cervical and rectal cancer [2]. Investigating the performance of APA in such cases would allow for a broader validation of the method.

Another aspect worth exploring is the influence of tumor size and geometry on the achievable thermal coverage. In this study, we have evaluated the approach on a small irregularly shaped deep-seated tumor in the H&N region, representing a challenging scenario for hyperthermia treatment planning. However, larger or more irregularly shaped tumor models could present different optimization challenges. As a future work, systematic testing on a variety of tumor shapes and volumes would help quantify the robustness and adaptability of the APA.

Finally, although we limited the comparison of the proposed algorithm to the PSO method—selected because it is widely used in clinically applied SAR-based optimization routines [23]—other optimization strategies could serve as relevant benchmarks. For instance, time reversal [22] has been proposed in the literature as an alternative field-focusing method. While time reversal relies on certain approximations and may not capture the full-wave nature of a meta-heuristic optimization approach like PSO, its comparative evaluation against APA in realistic patient models could provide further insights.

## 5. Conclusions

This study presented a deterministic framework for SAR-based optimization in microwave cancer hyperthermia, using the Alternating Projections Algorithm (APA) as an efficient alternative to conventional meta-heuristic approaches such as the particle swarm optimization (PSO). By enforcing a two-level power mask, APA effectively shapes the electromagnetic energy deposition, achieving both strong tumor targeting and suppression of hotspots in surrounding healthy tissues.

To guide the mask selection process, we introduced an adaptive threshold search strategy based on the defined $V_{\chi \%}^{H}$ metric, which quantifies off-target SAR exposure. This metric enabled a systematic trade-off analysis, allowing the APA method to dynamically balance tumor coverage with safety constraints, while ensuring efficient heating of the tumor target.

The methodology was validated in both a simplified phantom and a realistic H&N anatomical model. In both cases, the APA method achieved SAR distributions and temperature profiles comparable to those obtained with the PSO algorithm, but with a significantly reduced presence of hotspots and lower required input power. Specifically, in the simplified model, comparable heating was achieved with just 16 W for APA versus 26 W for PSO. Similarly, in the realistic setup, APA reached the desired thermal performance with only 44 W compared to 60 W for PSO.

In conclusion, the proposed APA-based SAR optimization method offers a robust and tunable alternative to PSO for SAR-based optimization in microwave hyperthermia treatment planning. Its deterministic nature and improved SAR and temperature distributions make it particularly attractive for anatomically complex targets, such as those found in the H&N region. Here, the algorithm was demonstrated using a two-level power mask; future work will explore its extension to multi-level masks and assess the resulting effects on focal quality and computational efficiency.

## Figures and Tables

**Table 1:** Optimized antenna excitation coefficients (amplitudes and phases) for APA and PSO.

*n*	APA Optimization		PSO Optimization	
	Amplitude (V)	Phase (°)	Amplitude (V)	Phase (°)
1	4.68	45.3	3.591	318.0
2	5.06	29.4	2.270	288.3
3	3.37	33.2	3.053	298.3
4	3.77	76.1	3.568	349.6
5	3.15	124.4	4.686	57.5
6	1.05	120.6	2.680	359.9
7	1.08	128.4	2.912	326.2
8	3.84	92.4	4.719	0.0

**Table 2:** Comparison of the convergence indicators for APA and PSO.

	APA Optimization	PSO Optimization
Num. iterations	1001	266
Total time (s)	128	140
HTQ (final)	0.8089	0.7998

**Table 3:** Standard thermal estimators obtained with the different methods.

Method	T90 (°)	T50 (°)	T10 (°)
PSO	42.62	43.14	43.48
APA	42.55	43.14	43.51
APA (finer mesh)	42.63	43.13	43.46

**Table 4:** Standard thermal estimators obtained with the different methods.

Method	T90 (°)	T50 (°)	T10 (°)
PSO	41.08	41.94	42.63
APA	41.05	41.83	42.50

## Data Availability

The original contributions presented in this study are included in the article. Further inquiries can be directed to the corresponding author.
